# Simulating and stimulating performance: developing a next-generation music performance simulator

**DOI:** 10.3389/fpsyg.2025.1694986

**Published:** 2025-10-31

**Authors:** George Waddell, Richard Bland, Aaron Williamon

**Affiliations:** ^1^Centre for Performance Science, Royal College of Music, London, United Kingdom; ^2^Creative Technologies, Royal College of Music, London, United Kingdom

**Keywords:** performance, simulation, experiential learning, distributed simulation, acoustic simulation, mixed reality, education

## Abstract

The spaces where musicians practice often differ considerably from where they perform. As such, musicians are an emblematic example of performers who must adapt their skillset to contexts where the visual, aural, social, and psychological environment introduces significant increases in variety, risk, and pressure. Numerous domains address this disparity through the use of simulation, giving the performer the opportunity to learn and challenge their skills in contexts more closely resembling real-world conditions. This article describes the implementation of simulation in music performance contexts through the development of the second generation of performance simulation technologies at the Royal College of Music. It outlines the design requirements for a large, immersive space with a high degree of flexibility in recreating the visual and acoustic atmosphere of a performance stage and corresponding backstage area, while also facilitating performance capture and analysis. Applications for such a facility are outlined to advance research, teaching, and knowledge exchange within and beyond music performance.

## 1 Introduction

Expert performers across domains spend considerable time developing and honing their physical, cognitive, social, and technical performance skills. The environments in which these skills are learned, however, are often radically different from those where they are performed. Study and practice spaces are generally plain, basic in character and variability, and present little scope to explore personal or professional risk. Conversely, performance environments are often significantly more varied, more provocative, and can expose the performer to consequential external pressures—competition, judgment, publicity—that may change reputational, financial, or competitive status and may impact their wellbeing. Where such pressures lead to decreased performance quality and consistency, this can also negatively impact those who rely on their performances: audiences, clients, patients, passengers, and so on. Expert performers, and those individuals and organizations who train and coach them, must therefore bridge the gap between the environments in which learning is undertaken and the conditions under which performance must occur.

An increasingly common tool in making this transition is performance simulation. By simulating risk and variability while providing control over the environment, sophisticated simulation tools have been used to train performers in the fields of aviation (Hamman, 2004), sport (Miles et al., 2012), firefighting (Bliss et al., 1997), medicine (Kneebone et al., 2010), public speaking (Slater et al., 1999), and more. While simulations can focus on a particular sense or modality, applications often blend a combination of sensory, environmental, and social elements that are created through a combination of virtual, physical, and theatric means, blurring the lines between the real and unreal while sometimes incorporating human actors to provide rich narrative and social cues (Kneebone et al., 2010). Simulation designs can put particular focus on a few key elements central to the experience, while factors in the periphery can be represented in simplified forms to reduce the complexity and expense of creating a simulation (Kassab et al., 2011). Similarly, simulations may focus only on replicating the specific technical skill being honed—a particular surgical technique, interaction with an aircraft control console, hitting a baseball—or may use simulation to place these skills within and prepare for the real-world contexts of, e.g., collaborative surgical theatres, weather-rattled cockpits, and arenas full of the opposition’s jeering fans. In contexts such as music or public speaking, there is generally no need to simulate the technical skill itself as the performers already have the tools at hand to execute it and therefore attention can be placed fully on simulating the environment.

In music, the sizeable gap between the practice rooms where musicians rehearse and the stages where they perform presents a particular challenge to which such tools of simulation can be applied. Musicians tend to spend a considerable amount of time practicing in relatively small spaces which are acoustically and visually unremarkable and involve little social interaction. While the environments where they might rehearse in groups or learn in one-to-one or classroom settings may be larger and more varied, there remains a marked difference between these and the venues where musicians must wait backstage in anticipation before walking out to lights, applause, and the scrutiny, expectation, and response of an audience or audition panel. This combination of heighted perceptions of expectation, evaluation, consequence, and lack of control over the situation can contribute to the high levels of Music Performance Anxiety found across music student and professional populations (see Herman and Clark, 2023 for a review). In such stressful situations, performers become less likely to commit to conscious, goal-directed action (Schwabe and Wolf, 2011). As such, the celebrated ‘flow’ state in which performers experience high levels of focus, self-awareness, self-confidence, immersion, enjoyment, and performance quality can be disrupted (Zielke et al., 2023; Tan and Sin, 2021). As musicians who report a sense of preparedness, control over the situation, high levels of self-efficacy, and flow experiences also report higher feelings of success on stage (Clark et al., 2014), practice opportunities are required that allow performers to develop the skills and perceptions of control within dynamic performance environments.

A significant step in blending virtual, physical, and social elements for a music-based simulation was developed in 2011 at the Royal College of Music (RCM) (Williamon et al., 2014). For the *Performance Simulator,* a virtual audience and a three-person audition panel were created by filming actors who produced a range of behaviors typically found, respectively, in a concert recital or performance examination context. The resulting audio and video recordings were compiled in a custom software interface, allowing an operator to trigger reactions ranging from boos to standing ovations among the audience and positive, neutral, or negative greetings, general viewing actions, and dismissals from the three-person panel, as well as distractions including ringing phones or spoken interruptions. This virtual world, presented via projection and speakers, was then surrounded by the physical environment of a small stage including curtains, spotlights, and a backstage area in which a performer would have to wait to perform. The virtual simulation extended to this backstage area via the sounds and simulated CCTV footage of a waiting audience or panel (Williamon et al., 2014). To link these experiences and provide further immersion into the simulated performance, the facility operator also played the role of the “backstage manager,” greeting and guiding the performer through the experience without alluding to its artificial nature. Studies of electrocardiographic and self-reported state anxiety data demonstrated that the simulation provoked anxiety responses comparable to a live audition (Williamon et al., 2014), with students perceiving the simulation to be an effective tool to provoke and train for performance anxiety (Aufegger et al., 2017). The simulation of the backstage as well as the onstage experience also allowed researchers to demonstrate how anxiety is present, and often peaks, during the pre-performance period (Chanwimalueang et al., 2017).

Subsequent applications of performance simulation for musicians have been constructed and have demonstrated reductions in self-reported performance anxiety (Choi et al., 2024; Bissonnette et al., 2015, 2016) and changes in performers’ onstage movement (Glowinski et al., 2015). Prototype music performance simulations have also been developed in virtual reality (VR) headsets (ThompSon et al., 2025; Bellinger et al., 2023; Bertsch and Peschka, 2023; Ppali et al., 2022; Orman, 2003, 2004) and for the training of music performance judges (Waddell et al., 2019). However, widescale implementation and testing of performance simulation for musicians remains to be carried out, as does incorporating technological improvements in immersive visual and acoustic environments. To advance the field of performance simulation, this article presents a next-generation performance simulation facility launched in 2024 at the RCM and sets out the routes by which such technology can be used to progress research, training, and knowledge exchange across performance domains.

## 2 Requirements of a next-generation performance simulator

Built upon over a decade of experimentation with and refinement of the original RCM *Performance Simulator*, the development team (i.e., the authors) set out key areas where a new approach could expand the simulator’s functionality and update it with state-of-the-art technologies. In 2024, two new simulation facilities were launched putting this into practice.^1^

### 2.1 Visual simulation

**Figure 1:**
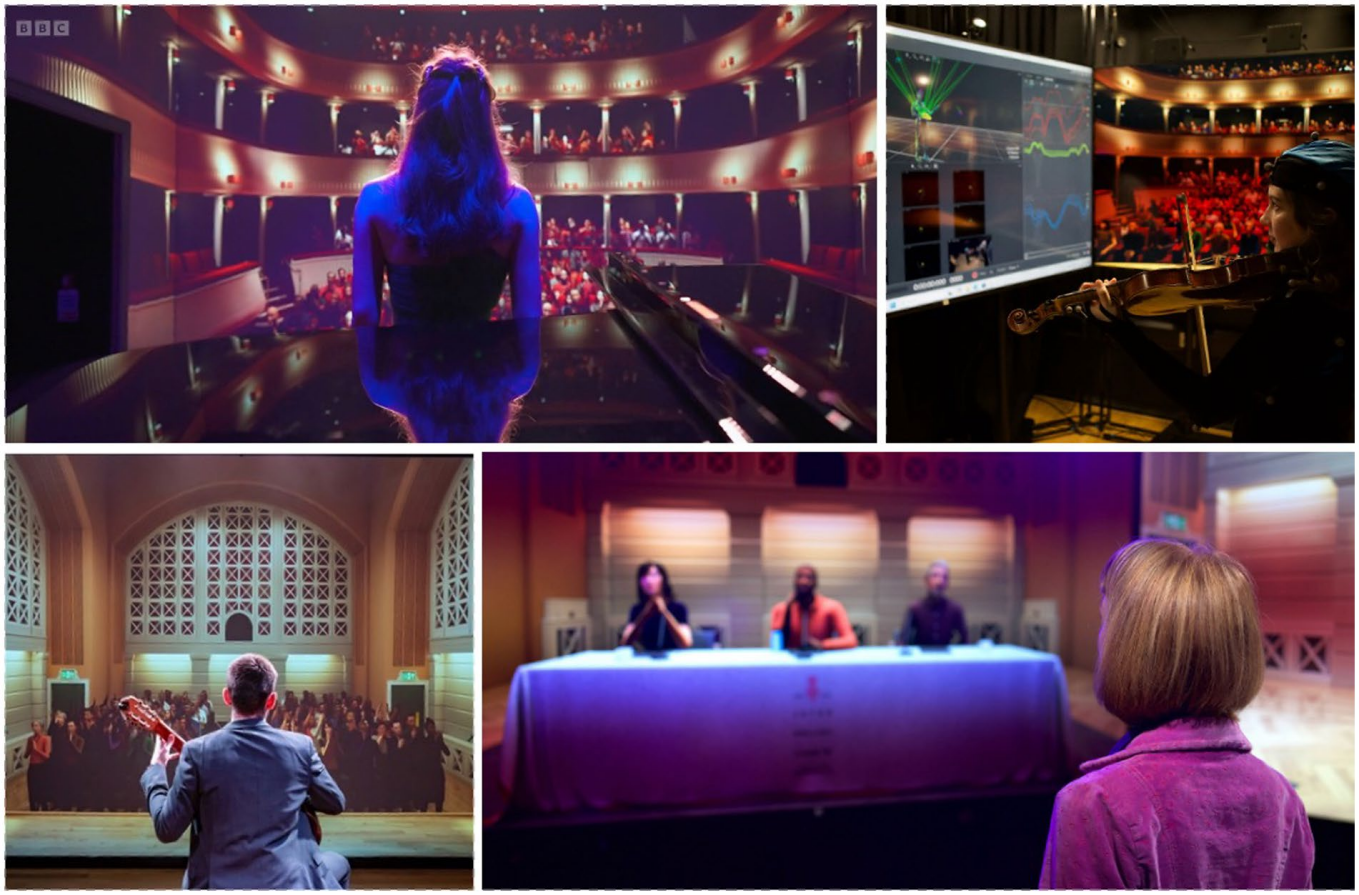
The *Performance Laboratory* is a next-generation training and research facility for practicing and studying performance. Clockwise from top left: a singer in a simulation of the RCM Britten Theatre (as seen on *BBC Click*, https://youtu.be/K0eMCV7vJg8); a violinist accesses motion capture data; a speaker delivering a business pitch to a simulated panel of judges; a guitarist in a simulation of the RCM Performance Hall.

While the use of pre-recorded actors offered enhanced visual realism of the original simulations, it limited the degree to which new people and behaviors could be introduced into the virtual display. Furthermore, the original audience and audition panel members were situated within a featureless black void, allowing for a performer to infer a larger space but limiting the degree to which the visual features of a concert stage could be presented. The new simulation makes use of fully virtual environments rendered within the Unreal Engine platform and created in collaboration with Ammonite Studios. This allows for high-fidelity recreations of actual or imagined spaces (e.g., three of the RCM’s performance venues were recreated using LIDAR scans of the corresponding three-dimensional spaces) in which the lighting can be adjusted and a variable number of seats populated with virtual avatars. Each virtual human is driven by real motion captured via motion tracking and voices recorded from actors to provide highly interactive audiences or audition panels that can provide enthusiastic support, lukewarm responses, or irritating distractions. As scenes are rendered in real time, behaviors can transition seamlessly without the need to splice prerecorded videos and can be continually updated with new personas, behaviors, and environments as well as improvements to the underlying infrastructure to enhance photorealism, framerate, and computational efficiency. These virtual environments are then displayed on three sides of the performer via floor-to-ceiling projections (see Figure 1).

Implementation within VR headsets was considered and a prototype developed. However, several inherent limitations of current generation headsets restrained expansion on this front: (1) their obscuring of the hands and musical instruments (including spatial distortion, inferior resolution, and limited ability to seamlessly blend real and virtual elements via pass-through Augmented Reality functionalities); (2) isolation of musicians from collaborators and instructors in the physical space; (3) the physical presence of the headsets interfering with instrument placement (e.g., the violin) or weight distribution of the head; and (4) restricted ability of the musician to move through a variable physical and acoustic space, including moving from backstage to performance areas. As such, a Mixed Reality (MR) approach was favored in which fully virtual rendered worlds are displayed within a physical environment.

### 2.2 Acoustic simulation

Musicians have highly developed aural capacities; therefore, it is unsurprising that they have been found not only to be acutely aware of their acoustic environments but to adjust their playing accordingly (Bolzinger et al., 1994; Berg et al., 2016). Given the high degree of variability in acoustic features of different performance spaces, let alone the difference between a performance space and a practice room, a fully immersive music performance simulation must be able to recreate (or at least approximate) a variety of acoustic experiences. These new simulations achieve this through virtual means: comprehensive acoustic treatment of the room removing characteristic reverberations and early reflections is combined with a sophisticated constellation system (via Meyer Sound) of 64 speakers, microphones, and a suite of processors that can recreate the acoustic nuances of specific venues (e.g., using the same scans of the RCM venues mentioned above, or a simulation created of the Royal Albert Hall) or of hypothetical venues (e.g., a cathedral or a “jazz club”) which can be recalled instantaneously by the performer or an operator.

### 2.3 Scope and variability of the physical environment

The size of the original performance simulator was limited to five onstage performers with another five observing or waiting backstage. To increase the range of possible performance experiences as well as increase pedagogical use cases, the largest simulation was built to accommodate ensembles of up to 20 onstage musicians as well as up to 40 observers. Concert-grade lighting rigs allow for variability of the lighting conditions, linked to the virtual world such that changes to the real-world lighting trigger corresponding changes in the virtual environment. The backstage area has been enhanced by the additions of variable lighting, the ability not only to hear but to view the audience in their seats before the performance, and the ability to trigger pre-recorded announcements of time to performance and calls to take the stage.

### 2.4 Performance capture

In addition to allowing performers to experience the physical, physiological, emotional, and social implications of live performance, simulations allow researchers to measure these effects with the benefits of controlled laboratory conditions. As such, the simulations incorporate broadcast-quality audio and video recording alongside optical motion tracking (see Figure 1), as well as portable devices for eye-tracking and physiological measurement. In combination with self-report and qualitative tools, performance can be captured before, during, and after each critical moment.

## 3 Use cases

Technologies and approaches that allow for simulators to recreate a wider variety of settings also increase the breadth of use cases. In the development described here, we focus on three categories of use: (1) research, (2) teaching, and (3) knowledge exchange.

In research applications, performance simulation offers the opportunity to study human performance in settings combining the experimental control of the laboratory with the ecological validity of live field research. While compromises must inevitably be made in finding a middle ground between the stage and the laboratory, simulated performance offers a space that can increase the rigor of research in the former and the generalizability of research in the latter. In this space, researchers can examine how the performance environment affects individuals’ physiology, psychology, cognition, and behavior, focusing on elements including performance anxiety, self-efficacy, communication, and performance quality. More broadly, musicians’ engagement with performance simulation can also be used to further knowledge of musicians’ evolving use of and attitudes toward technology (see Waddell and Williamon, 2019).

In teaching, performance simulation allows musicians and their teachers to focus on *practicing performing*, rather than practice that leads to performance. This can be applied at every stage in the pipeline; those approaching performance for the first time are able to use a controlled, risk-free environment to acclimatize themselves to the pressures of a stage, perhaps introducing elements such as enhanced lighting, acoustics, and audience behaviors one at a time until they are ready for the rich complexity of a live performance. Intermediate performers can use simulation to prepare for the leaps to larger venues and auditions or competitions of increasing consequence. Expert performers can hone their skills and ensure that they have optimized their performance for a particular environment while enhancing their ability to adapt spontaneously to unexpected challenges. Within the conservatoire, these simulations are being incorporated into teaching focusing on musicians’ wellbeing to help developing musicians build strategies to combat performance anxiety and thrive in onstage performance settings (Waddell et al., 2025). Simulation can also be used as part of a wider framework in which technology use more generally (e.g., the use of recordings) can further the principles of self-regulated learning by facilitating the evaluation, execution, and review of practice (Waddell and Williamon, 2024), joining the widening trend of technology’s use in enhancing how performance is learned and taught (for a review, see Ramirez-Melendez and Waddell, 2022).

Music performance simulation also opens a range of possibilities in exchanging knowledge beyond musical practice. In addition to recreating spaces familiar to those who regularly perform in them, simulations can help those outside of the domain better understand the experience of performance. Members of the public, educators, politicians, policymakers, and a wide range of professionals can experience music performance simulation to further their understanding of the challenges faced by performing artists and to help those working in the industry thrive in challenging settings. Simulation can also help individuals focus afresh on aspects of their own work. For instance, at the RCM, we have used the simulations to deliver communication and leadership training to business executives (Lane, 2025) and in exchanges with coaches and staff from the world of elite sport to consider the development of young talent on a global stage.

## 4 Discussion

Music performance joins an increasingly diverse range of domains employing simulation, from sport to medicine to aviation. While simulations of an aircraft or surgical facility will inevitably focus on the development of targeted technical skills, simulations of the stage must shift the focus to human interaction and how the eyes and ears of admirers and judges affect performers. The social pressure of performance is felt across a vast range of fields and professional contexts, with potential consequences for personal performance, educational outcomes, and health across local and global communities. As such, much work remains to integrate simulation more fully into performance education and ongoing professional development, with particular focus on accessibility, portability, and affordability. Further developments in VR and Augmented Reality (AR) headsets that move towards seamless blending of real-world, virtual, and social elements in devices that are increasingly lightweight and affordable will also increase the possible applications for organizations and personal use without the need for large-scale and expensive facilities. Advancements in hardware and software could have particularly significant impact for communities and individuals in relatively remote and socioeconomically deprived areas that do not have access to facilities in which they can experience and prepare for intensive performance environments. Of course, simulation is unlikely to replicate the full complexity, interactivity, and psychological immersion of live settings for those training to perform, and as such performance training should continue to blend didactic approaches with scaffolded real-world training opportunities guided by experienced practitioners and use simulation to bridge the gap between them. However, this gap will continue to narrow as evolving simulation technologies allow the practice environments themselves to take on more characteristics of the live environment and especially in cases where technology-driven performance environments, such as remote robotic surgeries or concerts streamed from broadcast studios, evolve to more closely resemble the tools used to simulate them. Advancements in Artificial Intelligence (AI) may help drive this by facilitating the creation of new environments and by increasing the automation and interactivity of the human elements within them, with virtual audiences and panelists who independently respond to what they see and hear. Furthermore, in societies and industries increasingly dominated by the performance of AI, it will become all more vital to identify, enhance, and celebrate the unique contributions of human creativity and performance. Those who have the tools to bridge the gap between their practice and performance will be better equipped to thrive in an increasingly uncertain world.

## Data Availability

The original contributions presented in the study are included in the article, further inquiries can be directed to the corresponding author.

## Footnotes

^1^The present article describes the larger of two performance simulation facilities. A second, smaller facility was constructed for the purpose of individual and small-group practice, training, and research that mirrors the functionality using high resolution LED displays, a simplified acoustic enhancement system, a 9-camera motion tracking system, and variable temperature control.
